# Imaging with ultrasound in physical therapy: What is the PT’s scope of practice? A competency-based educational model and training recommendations

**DOI:** 10.1136/bjsports-2018-100193

**Published:** 2019-04-25

**Authors:** Jackie L Whittaker, Richard Ellis, Paul William Hodges, Cliona OSullivan, Julie Hides, Samuel Fernandez-Carnero, Jose Luis Arias-Buria, Deydre S Teyhen, Maria J Stokes

**Affiliations:** 1 Department of Physical Therapy, Faculty of Rehabilitation Medicine, University of Alberta, Edmonton, Alberta, Canada; 2 Health and Rehabilitation Research Institute, School of Clinical Sciences, Auckland University of Technology, Auckland, New Zealand; 3 School of Health and Rehabilitation Sciences, The University of Queensland, Brisbane, Queensland, Australia; 4 Department of Physiotherapy and Performance Science, University College Dublin, Dublin, Ireland; 5 School of Allied Health Sciences, Griffith University, Brisbane, Queensland, Australia; 6 Departmento de Enfermeria y Fisioterapia, Universidad de Alcala de Henares, Madrid, Spain; 7 Departamento de Fisioterapia, Universidad Francisco de Victoria, Madrid, Spain; 8 Walter Reed Army Institute of Research, Silver Spring, Maryland, USA; 9 School of Health Professions and Rehabilitation Sciences, University of Southampton, Southampton, UK

**Keywords:** curriculum, education, professional issues, rehabilitation, sonography

## Abstract

Physical therapists employ ultrasound (US) imaging technology for a broad range of clinical and research purposes. Despite this, few physical therapy regulatory bodies guide the use of US imaging, and there are limited continuing education opportunities for physical therapists to become proficient in using US within their professional scope of practice. Here, we (i) outline the current status of US use by physical therapists; (ii) define and describe four broad categories of physical therapy US applications (ie, rehabilitation, diagnostic, intervention and research US); (iii) discuss how US use relates to the scope of high value physical therapy practice and (iv) propose a broad framework for a competency-based education model for training physical therapists in US. This paper only discusses US imaging—not ‘therapeutic’ US. Thus, ‘imaging’ is implicit anywhere the term ‘ultrasound’ is used.

## Background

Many physical therapists embrace ultrasound (US) imaging as a means to deliver precise and personalised rehabilitation. Since the first published use of US by physical therapists (1980),^1–5^ there have been three notable milestones in the evolution of US use by physical therapists; a series of commentaries^6–8^ and original research published after the first International Symposium on Rehabilitative Ultrasound Imaging (RUSI; hosted by the US Army-Baylor University Doctoral Programme in Physical Therapy, Fort Sam Houston, Texas, 2006),^9^ a networking session at the International Federation of Orthopaedic Manipulative Physical Therapists conference (Quebec City, Canada, 2012),^10^ and a second (although not affiliated) international symposium hosted by the Universidad Francisco de Vitoria and the Spanish Society of Ultrasound in Physiotherapy (Madrid, Spain, 2016).^11^ Despite these efforts, there remains considerable confusion and inconsistencies in terminology associated with physical therapist use of US due, in part, to the diversity of manners in which US is used across the profession. It is also clear that previously identified gaps related to scope of practice (*a statement describing physical therapy within the context of the regulatory environment and the evidence base for practice within a jurisdiction. Scopes of practices are dynamic and evolving in accordance with changes in the evidence base, policy and needs of service users*)^12^ and specialised training are growing.

**Figure 1:**
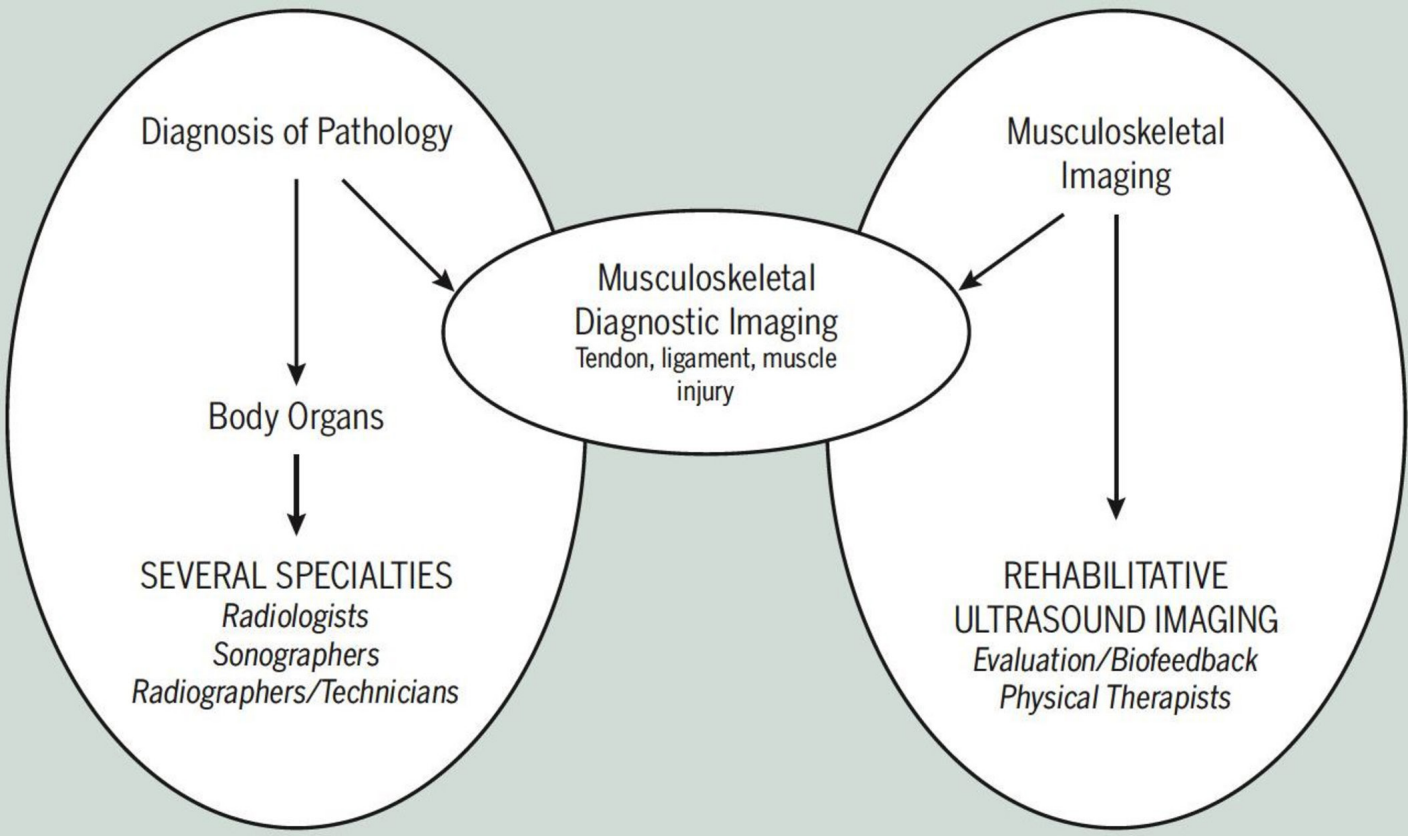
A visual representation of how the practice of RUSI evolved to fit into the larger field of medical us in 2006.^1 12^Reproduced with permission from the *J Orthop sports Phys ther*.

At the time of the 2006 symposium, the majority of reported uses of US by physical therapists involved the evaluation of muscle structure (morphology) and function, or as a source of biofeedback to aid rehabilitation of neuromuscular control. The term RUSI was coined to encompass these applications, and along with a definition (see below) an accompanying visual representation (figure 1) of how the practice of RUSI fits into the larger field of medical US was developed.

**Figure 2:**
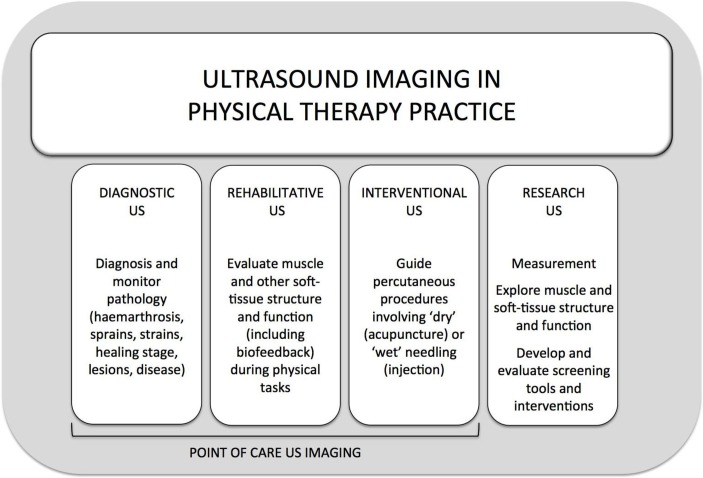
Current categories of US imaging use by physical therapists. US, ultrasound

Since 2006, three additional distinct categories of physical therapist use of US beyond RUSI have been identified. These applications include the following: diagnosing and monitoring pathology (diagnostic US); guiding percutaneous procedures involving ‘dry’ (eg, acupuncture) or ‘wet’ (eg, injection) needles (interventional US); and undertaking research (research US; see figure 2).

The three clinical categories (ie, rehabilitative, diagnostic and interventional US) of US use fall under the umbrella of ‘Point-of-Care Ultrasound’ defined as *an ultrasound examination performed by a qualified healthcare practitioner, usually as an adjunct to a physical examination, to clarify uncertain findings, or provide image guidance that improves the success and safety of procedures in the acute care setting, particularly when time saving for diagnosis or treatment is critical*.^13^ Point-of-care contrasts US evaluations performed in a dedicated imaging facility, or department, in a consultative process between the treating healthcare practitioner and a consulting imaging specialist. In the physical therapy context, point-of-care US can be defined as *a form of examination using US undertaken in a clinical practice setting with the intent of clarifying uncertain clinical examination findings to enhance the quality and effectiveness of a physical therapy intervention*. Given that physical therapy point-of-care US examinations fall within the scope of physical therapy practice and competence (knowledge, skills and abilities) of the examining therapist (as per the regulations of their jurisdiction), it is essential that it is understood that they are performed to direct a physical therapy intervention, not to provide a medical diagnosis or direct medical treatment.

Below, we define and describe the four broad categories of physical therapy US applications, discuss implications of the use of US by physical therapists on scope of practice and training, and propose a broad framework for a competency-based education model for training physical therapists in US use.

## Uses of US by physical therapists

This section proposes definitions and provides descriptions and examples of each of the four broad categories of physical therapy US applications outlined in figure 2.

### Rehabilitative US imaging

The most common uses of US by physical therapists reported in the literature fall within the realm of RUSI and have involved studies of the musculoskeletal system in a variety of settings (eg, sports medicine, orthopaedics, occupational, respiratory and pelvic health). Rehabilitative US was originally defined as *a procedure used by physical therapists to evaluate muscle and related soft tissue morphology and function during exercise and physical tasks…and to assist in the application of therapeutic interventions aimed at improving neuromuscular function.* 9 This includes measuring muscle morphology (eg, length, thickness, diameter, cross-sectional area, volume, fascicle length and penation angle)^14^; changes or differences in muscle morphology over time (eg, with ageing),^15^ between groups of people^16^ or with events (eg, contraction,^17^ injury,^18^ surgery,^19^ exposure to microgravity^20^; assessing the impact of muscle contraction on adjacent structures (movement and deformation of fascia,^21^ nerve,^22^ linea alba^23^ and visceral organs such as the bladder^8^ and urethra^24^; evaluating muscle composition^25^; and providing biofeedback.^26^ In the context of musculoskeletal and sports physical therapy, RUSI has been used to assess trunk muscle size and contraction to screen for injury risk,^27 28^ provide feedback and measure changes in muscle size as a result of injury prevention programmes^29^ or in response to conditioning^30^ or therapeutic interventions.^31^ In the context of pelvic health, RUSI has been used to understand,^8^ predict^32 33^ and manage urinary incontinence.^34^

### Diagnostic US imaging

Diagnostic US involves examining the effects of injury, lesion or disease on joint surfaces, muscle, tendon, ligament, bursa, vessels, nerves and solid visceral organs.^35^ Traditionally, these applications have fallen under the scope of a consulting imaging specialist (ie, radiologist or sonographer). Given that US is the most cost-effective, safe and rapid method of obtaining static and real-time images, many healthcare professions have embraced the technology for point-of-care applications. In the context of physical therapy, diagnostic US has been used to identify tendon abnormalities, to screen for tendinopathy risk,^36^ and assess humeral torsion or acromiohumeral distance in persons with rotator cuff pathology,^14^ haemarthrosis within the joints of persons with haemophilia,^37 38^ nerve excursion in entrapment neuropathy^39^ or ligament integrity after injury^40^ to inform rehabilitation. Although many physical therapists are appropriately trained in point-of-care diagnostic US, this application may be the most controversial given the potential overlap with other healthcare practitioners. A recent New Zealand survey highlighted that many physical therapists report confusion regarding their scope for diagnostic US applications.^41^

### Interventional US imaging

Interventional US involves using gray-scale brightness-mode (b-mode) US to accurately, efficiently and safely guide ‘dry’ and ‘wet’ needles for a variety of invasive interventions including acupuncture, dry needling, percutaneous electrolysis, injection or aspiration. US-guided needling and injections have been shown to be more accurate and efficacious than landmark-guided injections.^42^ Although physical therapy practice acts vary globally, in regions where therapists are allowed to use dry and wet needles, interventional US has been employed to safely guide dry needles for acupuncture,^43^ trigger point ‘release’,^44^ and percutaneous electrolysis (ie, application of mechanical stimulation and electric current through an acupuncture needle theorised to provide controlled microtrauma to stimulate tissue repair).^45 46^

### Research US imaging

US is used in basic, applied and clinical research that aims to inform physical therapy practice. For example, US has been used to improve our understanding of the impact of pain and injury on motor control^47^ and muscle morphology,^18^ and the relationship between motor control and function,^48^ to determine which patients may benefit from a specific treatment approach,^31^ and to enhance motor learning and treatment efficacy via augmented feedback.^49^ More sophisticated applications of US have been used to elucidate the mechanisms underlying dry needling techniques,^50^ measure the excursion of nerves with movement,^51^ assess the biomechanical parameters (ie, stiffness) of soft tissues^52 53^ and how this is changed by treatment,^54^ the dynamics of pelvic floor muscle contraction,^24^ and effectiveness of physical therapy interventions.^55^ Similar to image-guided interventions, US has been used for many years to guide insertion of intramuscular electromyography electrodes into muscles that are deep,^48^ small^56^ or associated with high risk (eg, diaphragm.^57^ Beyond these applications, there is a large body of literature assessing the reliability and validity of US for examining various muscles,^58–61^ and nerves,^22^ as well as the application of US into physical therapy practice.^62^

### US technologies and display modes

It is important to note that within each of the four categories of physical therapy US applications, a variety of US-based imaging techniques can be used depending on the clinical or research goal. For example, gray-scale b-mode and motion (m)-mode US may be used to measure the morphological characteristics of a muscle,^63^ identify boney changes associated with lateral epicondylalgia^64^ or guide an acupuncture needle.^45^ In contrast, real-time Doppler US allows for dynamic high-resolution evaluations of tendon neovascularity.^65^ Elastography enables the quantification of the biomechanical properties (ie, stiffness) of soft tissues (eg, muscle, tendon, ligament) and subsequently may have a role in assessing the effectiveness of physical therapy interventions^31 54^ or stages of tissue healing.^66^

## Implications for scope of practice, regulation and training

In addition to a lack of regulatory oversight, surveys conducted in the UK,^67^ Australia^68^ and New Zealand^41^ demonstrate that there is no internationally accepted curricula for physical therapists training in US, with continuing education or mentoring opportunities varying widely across countries, and no minimal competency required for using US for patient care. One explanation for these gaps is that unlike diagnostic and interventional US, RUSI is a relatively new application and one that sits almost entirely within the scope of the physical therapy profession (sports scientists, sport therapists and osteopaths also perform RUSI applications). Faced with the rapid growth of US use by physical therapists over the last decade, the profession is faced with a situation in which its traditional scope is being challenged to evolve. Clear and consistent guidance from regulatory and professional associations could assist in mitigating these gaps and confusion.

Each category of physical therapy US is associated with unique knowledge, skill sets and potential for perceived infringement with the scope of other healthcare practitioners. Although there is some foundational overlapping concepts, the issues and barriers associated with specialised training, competent use and reporting of these applications differ. In the fields of diagnostic and interventional US, there are established criteria for training, competent use and regulation, as outlined by the WHO,^69^ and international oversight from the World Federation for Ultrasound in Medicine and Biology. Physical therapists wanting to become skilled in the use of diagnostic and interventional US can access training through existing channels consistent with these standards. With that said, it is acknowledged that in some countries there may be limited access to these established training pathways afforded to physical therapists, and existing educational models may not include physical therapy-specific applications. It is also important to consider that the practice of physical therapists gaining their US training through courses established for other healthcare practitioners (eg, radiologists, sport and exercise medicine physicians, sonographers) may lead to physical therapists operating outside of their professional scope of practice due to an increased familiarity with non-physical therapy applications. There is a need for evidence-based diagnostic and interventional US training programme that meets the unique needs of physical therapists and highlights the issues associated with the scope of practice and licensing.

Beyond training, it is important to consider that although diagnostic and/or interventional US may fall within the scope of physical therapy (assuming suitable training is obtained) in some jurisdictions, for the majority this is not the case. Regardless of training or expertise, physical therapists should clarify their scope of practice for these US applications by contacting their regulatory body prior to performing diagnostic or interventional US. In many instances, a change in legislation to extend the scope of physical therapy practice in a jurisdiction may be required before therapists can use US in this manner.

In contrast to diagnostic and interventional US, and despite increasing evidence that demonstrates a role for RUSI in physical therapy, the field of RUSI lacks professional oversight, standard curriculum and regulation for training. These deficiencies have resulted in a paucity of high-quality, evidence-based training opportunities; a lack of standardisation in the performance and reporting of RUSI applications; and a potential for insufficiently trained operators.^41 67 68^

## A framework for US training for physical therapists

As competent use of US for point-of-care or research purposes is not part of an entry to practice skill set, and generally absent in physical therapy entry-to-practice education programme, access to postgraduate education to support safe competent practice is needed. The sections that follow contain key competencies, options for delivery and learning objectives for this training. This content is based on literature review, and the extensive experience of developing and delivering US training to physical therapists by the authors, in conjunction with consultation and collaboration with numerous medical and sonographic professionals and professional organisations (eg, the British Medical Ultrasound Society), over the last 30 years. The intent of this material is to provide a foundation for individuals and organisations developing or evaluating RUSI, diagnostic or interventional US courses for physical therapists.

### Core competencies for US use by physical therapists

The Canadian National Physiotherapy Advisory group defines an essential competency as *the repertoire of measurable knowledge, skills and attitudes required by a physical therapist throughout their professional career*.^70^ For physical therapists that use US in their practice, this includes the knowledge, skills and attitudes associated with safe, competent conduct and interpretation of US examinations. Fundamental competencies that span all uses of US by physical therapists and those unique to RUSI, diagnostic, interventional or research US examinations are outlined in Box 1.

Box 1. Summary of fundamental competencies (knowledge, skills and attitudes) for safe and efficacious use of US by physical Therapists*

**Fundamental Knowledge, Skills, Attitudes**

- Professional and ethical considerations
- Communication
- Basic anatomy and physiology
- US basic physics
- US safety, upkeep and hygiene
- Basic US terminology and instrumentation
- Basic US image generation and optimisation
- Basic US interpretation including artefact

**RUSI Competencies Knowledge, Skills, Attitudes**

- Physical therapy scope and history of RUSI
- Detailed anatomy and physiology
- Theoretical foundations of neuromuscular function and dysfunction
- RUSI terminology and instrumentation
- RUSI image generation and optimisation
- RUSI interpretation
- Special issues for specific body regions and applications
- Integration of RUSI findings for prevention and management of clinical conditions
- Evaluate the use of RUSI in clinical practice

**Diagnostic US Knowledge, Skills, Attitudes**

- Physical therapy scope and history of diagnostic US
- Detailed anatomy and physiology
- Theoretical foundations of pathoanatomical and biopsychosocial models of pain
- Diagnostic US terminology and instrumentation
- Diagnostic US image generation and optimisation
- Diagnostic US interpretation
- Integration of diagnostic US for prevention and management of clinical conditions
- Evaluate the use of diagnostic US in clinical practice

**Interventional US Knowledge, Skills, Attitudes**

- Physical therapy scope and history of interventional US
- Detailed anatomy and physiology
- Interventional US safety
- Interventional US needle guidance principles, methods and accuracy
- Interventional US terminology and instrumentation
- Interventional US image generation and optimisation
- Interventional US interpretation
- Integration of interventional US for prevention and management of clinical conditions
- Evaluate the use of interventional US in clinical practice

**Research US Knowledge, Skills, Attitudes**

- History of physical therapy research using US
- Relevant anatomy and physiology
- Research context background knowledge
- Study design and research methodology
- Research US methodology and approaches
- Research US ethics and safety
- Research US terminology, instrumentation and applications
- Research US image generation and optimisation
- Research US interpretation
- Research US dissemination

*It is recommended that all physical therapists that use US meet the fundamental competencies followed by one of the application specific competencies.RUSI, rehabilitative ultrasound imaging; US, ultrasound.

### Delivery format

Given that physical therapists who utilise US must demonstrate common fundamental and application-specific competencies, a competency-based education model of training is suggested. Competency-based education is driven by the ‘product’ rather than the process,^71 72^ whereby learning outcomes are first identified and the curriculum is built in discrete ‘steps’ to ensure that students achieve the competencies described in the learning outcomes. In the case of US, ‘steps’ could take the form of an ‘introductory’ (ie, fundamental knowledge and proficiency) module followed by completion of one, or several, ‘application-specific’ modules (ie, RUSI, diagnostic or interventional). The delivery of each module could take the form of didactic and/or practical instruction with each culminating in a practical examination of safety, technical aspects, and image generation and interpretation competence. This approach allows flexibility for the addition of future US applications and could be supplemented with formal or informal mentorship, supervision and case-based examination. In addition to instruction by physical therapists who are experts in this field, training should, where possible, involve other imaging disciplines (eg, sonographer/radiologist/interventional radiologists) and focus on the pathologies and disorders that physical therapists treat. Furthermore, it is important to consider that training could be provided in many settings (eg, entry and post-professional level) and through different delivery mechanisms (eg, pre-reading and exams, online resources, practical courses, virtual mentoring and supervised scanning or review of stored images or real-time clips for quality assurance, etc). There may also be value in embedding training within existing coursework in entry-to-practice programme (eg, electrophysical agents, anatomy, orthopaedics, neurology, professional issues courses or yearly or programme-end capping exercises).

### Curriculum

The competent conduct and interpretation (including background knowledge) of US examinations vary by the level of operator skill (eg, introductory vs advanced) and application (eg, RUSI, diagnostic, interventional, research). Suggested learning outcomes for ‘introductory’ and ‘application’ modules or courses are outlined in online supplementary table 2 located in.

10.1136/bjsports-2018-100193.supp1 Supplementary data

## Recommendation and future directions

Future efforts should focus on developing international standards for self-governance of US use by physical therapists and ensuring that training and practice standards are identified, reached and maintained. Failure to do this may result in restricted use of US by physical therapists in various jurisdictions. Greater interprofessional exposure to the use of US by physical therapists is needed to avoid inaccurate assumptions about professional infringement and to foster understanding of the unique applications of US that occur within physical therapy practice. Finally, it is imperative that physical therapists continue to provide evidence that US enhances the quality, effectiveness (including cost) and efficacy of physical therapy management.
