# Supplementary material for: Imaging with ultrasound in physical therapy: What is the PT’s scope of practice? A competency-based educational model and training recommendations
Source: Br J Sports Med. 2019 Apr 25;53(23):1447–53. doi: 10.1136/bjsports-2018-100193 (PMC6900235; doi:10.1136/bjsports-2018-100193)
Supplement: Supplementary data [file bjsports-2018-100193supp001.pdf]

TABLE 2: Suggested Competencies for Introductory and Application-Specific Modules for Physical Therapy Ultrasound Imaging Training

Introductory Module – Fundamental Knowledge, Skills, Attitudes Learning Outcomes

1. Demonstrate an understanding of professional and ethical considerations for the use of US in physical therapy practice
   - *Scope and code of physical therapy practice*
   - *Overview of the types and roles of current categories of US applications for musculoskeletal physical therapy: RUSI, Diagnostic, Interventional, Research*
   - *Patient consent: including limited scope of Point-of-Care applications*
   - *Storage of data*
   - *Convention for dealing with abnormal findings*
2. Demonstrate effective communication and team working skills
   - *Other healthcare practitioners*
   - *Patients and their caregivers*
   - *Third parties*
3. Demonstrate knowledge and understanding of basic anatomy and physiology *(pre-requisite)*
4. Demonstrate an understanding of physics principles relevant to US
   - *Piezo-electric and reverse piezo-electric effect*
   - *Sound wave propagation and echo production*
   - *Attenuation and acoustic impedance*
   - *Positional information and brightness*
5. Demonstrate knowledge, understanding and application of US safety, upkeep and hygiene standards
   - *Practicing and commenting beyond competencies and professional scope*
   - *Thermal and mechanical effects of US*
   - *As Low as Reasonably Achievable (ALARA) Principal*
   - *Infection prevention and control: USI transducer cleaning and disinfection (for intact skin, endocavity, and mucous membrane contact), indications for sterile gel use (i.e., mucous membranes or body fluid contact), and offset pad cleaning and disinfection*
   - *Ultrasound system and transducer maintenance*
6. Demonstrate an understanding of conventional US terminology and instrumentation:
   - *General US unit navigation and 'knobology'*
   - *Transducers: linear, curvilinear, vector, small parts, intravaginal/rectal, 3D, 4D*
   - *Definition, indications and limitations of b-mode, m-mode, panoramic, Doppler, shear-wave elastography display modes*
   - *Image manipulation functions: planes, depth, field of view, power, gain, focal points*
7. Apply basic skills to generate and optimize RUSI, Diagnostic and Interventional US images
   - *Transducer selection: frequency, resolution and field of view*
   - *Transducer location and orientation, including slide, tilt, rotation, heel-toe probe motion*
   - *Imaging technique: coupling agent, transducer pressure, incidence angle and indications for offset pads*
   - *Image optimization: frequency, depth, power, gain, focal points and position*
   - *Ergonomics*
   - *Real-time imaging*
8. Demonstrate a basic ability to interpret and evaluate US images
   - *Anatomical orientation*
   - *Echogenicity*

Whittaker JL, *et al. Br J Sports Med* 2019; 53:1447–1453. doi: 10.1136/bjsports-2018-100193

- *Tissue differentiation: fluid, muscle, tendon, ligament, fascia, vessel, nerve, cartilage, bone*
- *Image search patterns*
- *Challenges associated with interpreting 2D and real-time studies*
- *Introduce quantitative measurement highlighting the need for standardization*
- *Basic artifacts: acoustic enhancement, acoustic and edge shadow, and twice-around*

RUSI Module – Knowledge, Skills, Attitudes Learning Outcomes*

1. Demonstrate detailed knowledge and understanding of physical therapy scope of practice and history of RUSI
   - *Rationale for RUSI*
   - *Physical Therapy RUSI scope of practice*
   - *Detailed examples of RUSI: include trunk, upper and lower quadrants as appropriate*
2. Demonstrate detailed knowledge and understanding of anatomy and physiology *(pre-requisite)*
3. Demonstrate advanced knowledge and understanding of the theoretical foundations of neuromuscular function and dysfunction *(pre-requisite)*
4. Explain RUSI terminology and instrumentation
   - *RUSI definition and context*
   - *Imaging mode and display mode selection*
5. Apply advanced skill in RUSI image generation and optimization
   - *Transducer selection*
   - *Transducer location and orientation*
   - *Imaging technique: minimizing transducer motion during real-time studies*
   - *Image optimization: techniques for enhancing muscle boundaries*
6. Interpret and evaluate RUSI studies
   - *Static studies: anatomical features and, muscle and other soft-tissue composition, integrity and morphology*
   - *Measuring morphology: cross-sectional area, length, thickness, volume, angle*
   - *Measuring and interpreting echogenicity: implications for tissue quality*
   - *Real-time studies: muscle or other soft-tissue integrity, change in muscle morphology*
   - *Interpreting morphological changes of muscle: implications for muscle activity including the non-linear relationship between muscle activity and morphological changes, impact of contraction type and limitations*
   - *Measurement concepts: validity, reliability, minimal clinically important difference*
   - *Limitations of RUSI and inaccurate interpretations*
7. Discuss special considerations for RUSI of specific body regions
   - *Cervical, thoracic and lumbar spine*
   - *Chest, diaphragm and abdominal wall*
   - *Pelvic floor and bladder*
   - *Upper and lower extremity*
8. Discuss special considerations for specific RUSI applications
   - *Joint motion*
   - *Pelvic floor assessment (2D, 3D and 4D applications)*
   - *Diaphragm and breathing*
   - *Fascial motion*
   - *Nerve motion*
9. Apply clinical knowledge, reasoning and skills to integrate RUSI findings in the evidence-based prevention and management of clinical conditions

Whittaker JL, *et al*. *Br J Sports Med* 2019; 53:1447–1453. doi: 10.1136/bjsports-2018-100193

- *Risk prediction*
- *Assessment*
- *Guidance for intervention selection/targeting*
- *Education*
- *Biofeedback*

10. Evaluate the use of RUSI in clinical practice with reference to scientific research evidence

Diagnostic US Module – Knowledge, Skills, Attitudes Learning Outcomes*

1. Demonstrate detailed knowledge and understanding of physical therapy scope and history of diagnostic US
   - *Rationale for Diagnostic US by physical therapists*
   - *Physical Therapy Diagnostic US scope of practice*
   - *Detailed examples of Diagnostic US: include trunk, upper and lower quadrants as appropriate*
2. Demonstrate detailed knowledge and understanding of anatomy and physiology *(pre-requisite)*
3. Demonstrate advanced knowledge and understanding of theoretical foundations for pathoanatomical and biopsychosocial models of pain in musculoskeletal disorders *(pre-requisite)*
4. Explain diagnostic US terminology and instrumentation
   - *Diagnostic US definition and context*
   - *Imaging mode and display mode selection*
5. Apply advanced skill in Diagnostic US image generation and optimization
   - *Transducer selection*
   - *Transducer location and orientation*
   - *Imaging technique: minimizing transducer motion with real-time studies*
   - *Image optimization; techniques for enhancing differentiation of various media*
6. Interpret and evaluate Diagnostic US studies
   - *Pathology specific concepts for image acquisition and interpretation*
   - *Static studies: advanced tissue differentiation, trauma and tissue integrity, healing stages and pathology*
   - *Real-time studies: musculoskeletal tissue integrity and motion*
   - *Advanced artifact identification: anisotropy etc.*
   - *Region and application specific search patterns*
   - *Region and application specific quantitative measurement*
   - *Measurement concepts: standardization, reliability and validity*
7. Apply clinical knowledge, reasoning and skills to integrate Diagnostic US findings in the evidence-based prevention and management of clinical conditions
8. Evaluate the use of Diagnostic US in clinical practice with reference to scientific research evidence

Interventional US Module – Knowledge, Skills, Attitudes Learning Outcomes*

1. Demonstrate detailed knowledge and understanding of physical therapy scope and history of interventional US
   - *Rationale for Interventional US by physical therapists*
   - *Physical therapy Interventional US scope of practice*
2. Demonstrate detailed knowledge and understanding of anatomy and physiology *(pre-requisite)*
3. Demonstrate advanced skill in needling technique *(pre-requisite)*

Whittaker JL, *et al. Br J Sports Med* 2019; 53:1447–1453. doi: 10.1136/bjsports-2018-100193

- *Dry needing, percutaneous electrolysis, injection etc.*
- *Risks and ethics for needling/skin penetration*

4. Demonstrate and apply knowledge and understanding of Interventional US safety and hygiene standards and procedures
   - *Universal precautions*
   - *Indications for sterile gel*
   - *First aid protocol including instances of pneumothorax and vasovagal response*
5. Explain Interventional US terminology and instrumentation
   - *Needle optimization software*
   - *Power color-Doppler*
   - *Shear-wave elastography*
6. Apply advanced skill in Interventional US image generation and optimization
   - *Transducer selection*
   - *Transducer location and orientation*
   - *Imaging technique (initially on a phantom followed by a human model): skill development for coordination of needle and transducer motion, estimation of needle orientation prior to insertion, use of a needle guide, free hand insertion, identification of needling path to avoid specific structures (e.g., nerve, vessel, lung)*
   - *Image optimization: techniques for enhancing differentiation of various media including needle and trigger points*
7. Demonstrate advanced skill in interpretation and evaluation of Interventional US
   - *Static studies: neovascularity, tissue stiffness, heterogeneity index, histogram analysis*
   - *Real-time studies: needle manipulation*
   - *Region and application specific search patterns*
   - *Region and application specific quantitative measurement*
   - *Measurement concepts: standardization, reliability and validity*
8. Apply clinical knowledge, reasoning and skills to integrate Interventional US findings in the evidence-based prevention and management of clinical conditions
9. Evaluate the use of Interventional US in clinical practice with reference to scientific research evidence

Research US Module – Knowledge, Skills and Attitudes Learning Outcomes*

1. Demonstrate knowledge and understanding of the history of physical therapy research using USI
2. Demonstrate detailed knowledge and understanding of relevant anatomy and physiology *(pre-requisite)*
3. Demonstrate detailed knowledge and understanding of the relevant research context *(pre-requisite)*
4. Demonstrate detailed understanding of principles of study design and research methodology *(pre-requisite)*
5. Integrate USI procedures and approaches into research design and methodology
   - *Transducer, imaging mode and display mode selection*
   - *Considerations for synchronizing US signal with events or other signals*
   - *Considerations for data (longitudinal) collection*
   - *Pilot testing*
   - *Image analysis: on-machine vs. custom software signal post-processing, image scaling, image manipulation and standardization*
6. Apply standards of research ethics and safety principles during research using US
   - *Ethics considerations: non-ionizing radiation and considerations for intramuscular*

Whittaker JL, *et al*. *Br J Sports Med* 2019; 53:1447–1453. doi: 10.1136/bjsports-2018-100193

electrode insertion
- *Informed consent*

7. Explain Research US terminology, instrumentation and applications
   - *Basic imaging modes: definition, limitations and controls for b and m-mode*
   - *Advanced applications: definition, limitations and controls for Doppler, shear wave elastography, intramuscular electrode guidance, 3D/4D imaging*
8. Demonstrate advanced skill in Research US generation and optimization *(research question specific)*
   - *Transducer location and orientation*
   - *Imaging technique: controlling transducer motion*
   - *Image optimization: techniques for enhancing differentiation of various media*
9. Interpret and evaluate Research US studies *(research question specific)*
   - *Measurement concepts: standardization, validity, reliability, standard error, statistical vs. clinical significance*
   - *Interpretation of static studies: search patterns and basic quantitative measurements (e.g., width, cross-sectional area, angle etc.)*
   - *Interpretation of real-time studies: distinction between change in muscle size and muscle activity*
   - *Limitations: what US can and cannot be used for, caution when interpreting muscle activity and causes of inaccurate interpretation*
10. Demonstrate knowledge and understanding of the importance of dissemination of findings from research studies using US
    - *Radiological convention for orientation*
    - *Standardized terminology and reporting of methods and limitations*

**It is recommended that all Physical Therapists that employ US meet the fundamental competencies followed by one of the application specific competencies. The content of the RUSI, Diagnostic and Interventional Modules can be tailored to different regions of the body (e.g., cervical, thoracic or lumbar spine, upper or lower extremity) depending upon the scope of the training.*

b – brightness, m – motion, RUSI – Rehabilitative Ultrasound Imaging, US – Ultrasound Imaging

v
